# Oestrogen is important for maintenance of cartilage and subchondral bone in a murine model of knee osteoarthritis

**DOI:** 10.1186/ar3148

**Published:** 2010-10-05

**Authors:** Yvonne H Sniekers, Harrie Weinans, Gerjo JVM van Osch, Johannes PTM van Leeuwen

**Affiliations:** 1 Department of Orthopaedics, Erasmus MC, University Medical Center, 's Gravendijkwal 230, 3015 CE Rotterdam, The Netherlands; 2 Department of Internal Medicine, Erasmus MC, University Medical Center, 's Gravendijkwal 230, 3015 CE Rotterdam, The Netherlands; 3 Department of Otorhinolaryngology, Erasmus MC, University Medical Center, 's Gravendijkwal 230, 3015 CE Rotterdam, The Netherlands

## Abstract

**Introduction:**

Oestrogen depletion may influence onset and/or progression of osteoarthritis. We investigated in an ovariectomized mouse model the impact of oestrogen loss and oestrogen supplementation on articular cartilage and subchondral bone in tibia and patella, and assessed bone changes in osteoarthritis development.

**Methods:**

C3H/HeJ mice were divided into four groups: sham-operated, oestrogen depletion by ovariectomy (OVX), OVX with estradiol supplementation (OVX+E) and OVX with bisphosphonate (OVX+BP). Each mouse had one knee injected with low-dose iodoacetate (IA), and the contralateral knee was injected with saline. Cartilage was analysed histologically 12 weeks postsurgery; bone changes were monitored over time using *in vivo *micro-computed tomography.

**Results:**

In tibiae, OVX alone failed to induce cartilage damage, but OVX and IA combination significantly induced cartilage damage. In patellae, OVX alone induced significant cartilage damage, which was enhanced by IA. In both tibiae and patellae, OVX in combination with IA significantly decreased subchondral cortical thickness in an additive manner. OVX+E and OVX+BP inhibited tibial and patellar subchondral cortical thinning, inhibited patellar and tended to diminish tibial cartilage damage. In patellae, IA interacted with BP, leading to increased subchondral cortical and trabecular bone.

**Conclusions:**

This study demonstrates the significance of oestrogen for articular cartilage and subchondral bone and maintenance of healthy joints, supporting an etiological role for altered oestrogen signaling in osteoarthritis either by directly affecting cartilage or increasing susceptibility for an osteoarthritis trigger. The data strongly support the concept of involvement of subchondral bone plate in osteoarthritis.

## Introduction

Osteoarthritis (OA) of the knee is a common, disabling and expensive disease [1,2]. The knee is a complex joint consisting of three compartments: medial and lateral tibiofemoral joint (TFJ) compartments and the patellofemoral joint (PFJ). Although clinical studies of osteoarthritis of the knee have tended to focus on the tibiofemoral compartment alone, the patellofemoral compartment is often affected as well. An investigation in people with knee pain revealed that the most common radiographic pattern is combined TFJ and PFJ disease (40%), followed by isolated PFJ OA (24%) [3]. Isolated TFJ OA occurred in only 4% of subjects [3]. The patellofemoral joint is an important source of symptoms associated with knee OA, such as pain, stiffness and disability [4].

It has been suggested in the literature that oestrogen depletion plays a role in the onset or progression of OA. Men are known to have a higher prevalence of OA than women before the age of 50 [5], but after this age the prevalence is higher in women [6,7]. The prevalence increases with age in both men and women, but in women, it increases dramatically around the age of 50 [5,8,9], which coincides with menopause.

Also, in animal models, a link between oestrogen and OA has been found. A number of animal studies have been performed to investigate the effect of oestrogen depletion and oestrogen replacement on articular cartilage (reviewed in [10]). In several animal models, ovariectomy leads to osteoarthritic changes, and oestrogen replacement therapy reduces cartilage degradation [11-13]. Oestrogen acts via oestrogen receptors (ERs), which have been found in articular cartilage, bone, synovial tissue and ligaments [14-17], which may all be involved in OA. Several studies have reported associations between OA and polymorphisms in ERα and ERβ [18-20]. Also, low serum estradiol levels have been found to be associated with OA [21]. Taken together, these findings argue for a role of oestrogen in the development of osteoarthritis. However, patella OA has not yet been included in studies on oestrogen and OA.

The exact mechanism by which oestrogen affects OA is not known. Apart from a direct effect of oestrogen on cartilage, bone may also be involved. Oestrogen is known to affect bone metabolism and to regulate the balance between bone formation and resorption [22]. Subchondral bone changes have been reported in OA patients [23-25] and in animal models for OA [26-28]. It has been suggested that subchondral bone changes are important in the aetiology of OA [29]. Alteration in subchondral bone remodelling, and subsequently in bone structure, may lead to changes in load distribution. This may in turn cause or accelerate cartilage damage. Therefore, bone changes induced by oestrogen depletion may play a role in OA development.

Although the prevalence increases after the age of 50, not all postmenopausal women get osteoarthritis [30], indicating that hormonal changes alone are not enough to cause OA. We hypothesize that oestrogen depletion increases the susceptibility of tissues in the joint for changes, but an additional trigger is needed to develop osteoarthritic changes. This concurs with the idea that OA is a multifactorial disease. We addressed this hypothesis by investigating bone and cartilage changes in the proximal tibia and patella of knee joints of ovariectomized mice and ovariectomized mice receiving oestrogen replacement, combined with a mild osteoarthritis trigger induced by iodoacetate, an inhibitor of glycolysis that is an accepted model for osteoarthritis [31-34]. The impact of bone changes was investigated by using bisphosphonates to inhibit bone resorption after ovariectomy and iodoacetate.

## Materials and methods

### Animals

Female C3H/HeJ mice (Jackson Laboratories, Bar Harbor, ME, USA) were chosen because of their substantial bone loss after OVX [35]. Four animals were housed per cage and fed *ad libitum*. At 12 weeks of age, mice were randomly allocated to a treatment group (as explained below). At 24 weeks of age, the experiment was finished, and serum, knee joints and uteri were collected. The experiment was approved by the animal ethics committee DEC consult.

### Ovariectomy, oestrogen supplementation and bisphophonate treatment

All animals received a subcutaneous injection of buprenorphine (Temgesic; 0.05 mg/kg body weight) as an analgesic before the operation. Eight animals underwent sham ovariectomy (Sham), and 32 animals were bilaterally ovariectomized (OVX) to induce oestrogen depletion. Of the 32 OVX animals, 8 animals received estradiol supplementation and 8 animals were treated with the bisphosphonate alendronate. Estradiol was supplemented by subcutaneous implantation of an oestrogen pellet (Innovative Research of America, Sarasota, FL, USA) (OVX+E). This pellet continuously released 17β-estradiol at a rate of 12 μg/day. Bisphosphonate treatment was performed by weekly intraperitoneal injections of alendronate dissolved in saline (2 mg/kg body weight; donated by Merck, Whitehouse Station, NJ, USA) (OVX+BP). After OVX or sham, all mice received an intra-articular injection with 6 μl of 0.5% iodoacetate (IA, Sigma-Aldrich, St. Louis, MO, USA) in one knee, and 6 μl of saline (Sal) in the contralateral knee by an experienced researcher according to the protocol described by van der Kraan *et al. *[31]. This gave us the following experimental groups: Sham+Sal, Sham+IA, OVX+Sal, OVX+IA, OVX+E+Sal, OVX+E+IA, OVX+BP+Sal and OVX+BP+IA. In the OVX+E group, four mice died before the end of the experiment. The cause of this is unclear, but it has been shown that long-term estradiol supplementation causes death in an ovarian atrophy model in APPswe transgenic mice [36] as well as in a myocardial infarction model in C57BL/6J mice [37].

### Micro-CT analysis

Mice were scanned using an *in vivo *micro-computed tomography (micro-CT) [38,39] scanner (Skyscan 1076; Skyscan, Kontich, Belgium) at 9-μm voxel size. The mice were anaesthetized using a 5% isoflurane/oxygen mixture. Hindlimbs were stretched, taped to a polystyrene foam block and placed in a perspex holder to image both knee joints simultaneously without interference of abdominal tissues or tail and without needlessly radiating the abdomen. Mice were scanned every 3 weeks starting just prior to OVX or Sham operation (*t *= 0).

**Figure 1:**
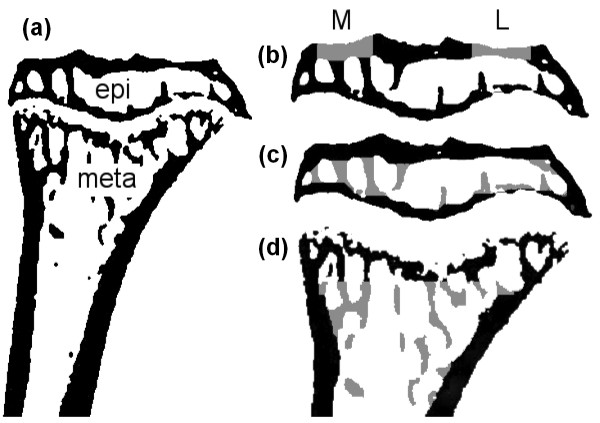
**Regions that were analyzed using micro-CT**. **(a) **Cross-sectional image of proximal tibia, including epiphysis (epi) and metaphysis (meta). **(b) **Epiphysis with medial (M) and lateral (L) subchondral plate depicted in light grey. **(c) **Epiphysis with trabecular bone depicted in light grey. **(d) **Metaphysis with trabecular bone region depicted in light grey.

Reconstructed grey-scale images were aligned visually using anatomical landmarks. The scans were segmented using a local thresholding algorithm [40], and the proximal tibia was isolated (Figure 1a). Using 3-D data analysis software (CTAnalyzer; Skyscan) the tibial epiphysis was selected as a region of interest for further analysis. Care was taken not to include any osteophytes. The epiphysis was further divided into a cortical and trabecular part [27], which were analyzed separately using a 3-D calculator (freely available [41]).

In the cortical compartment of the epiphysis, regions of interest (0.5 mm in mediolateral direction, 0.7 mm in anteroposterior direction) were selected at the middle of both the medial and lateral plateaus, representing the subchondral plate [28] (Figure 1b) to calculate 3-D thickness. To describe the bone structure of the epiphyseal trabecular compartment (Figure 1c), we calculated bone volume fraction, describing the ratio of bone volume over tissue volume (BV/TV). In the metaphysis, a region of interest (1 mm high), containing only trabecular bone (Figure 1d) was selected to calculate BV/TV.

To follow bone changes over time within one mouse, data sets of *t *= 0 and *t *= 12 were matched by rotating and translating one data set with respect to the other [38,39]. Registration (matching) software was used, which automatically matches two data sets using an optimization criterion based on maximizing mutual information [42].

**Figure 2:**
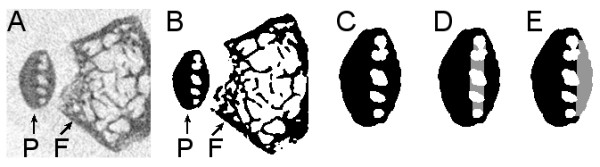
**Processing micro-CT data sets illustrated by one cross section**. **(a) **Reconstruction and alignment. P indicates patella, F indicates femur. **(b) **Segmentation. P indicates patella, F indicates femur. **(c) **Isolation of patella. **(d) **Separation of trabecular bone (grey) and cortical bone (black). **(e) **Region of interest of subchondral cortical bone (in light grey).

In addition to the proximal tibia, the entire patella was selected as a region of interest (Figure 2). The patella was further divided into a cortical part and a trabecular part (Figure 2d), which were analyzed separately. In the cortical compartment of the patella, a region of interest was selected containing cortical bone in contact with the articular cartilage of the patella (subchondral cortical bone; Figure 2e). The size of this region was 0.50 mm in the mediolateral direction and 0.97 mm in the proximal-distal direction. For this region, 3-D thickness was calculated. For the trabecular compartment, bone volume fraction, which describes the ratio of bone volume over tissue volume (BV/TV), was calculated.

### Histological analyses

Knee joints were fixed in 4% formalin, decalcified with ethylenediaminetetraacetic acid and embedded in paraffin. Frontal sections (6 μm thick) were stained with safranin O. Glycosaminoglycan depletion was assessed by loss of safranin O staining. The severity and extent of cartilage erosion in medial and lateral tibia plateaus and in patella were scored by a blinded observer using the grading and staging scoring system described by Pritzker *et al. *[43]. Per area, the average of three sections (100 μm apart) was determined, with a maximum score of 24 for each area.

The presence or absence of osteophytes was scored both on histology and on micro-CT scans. Osteophytes visible on histology can be either purely cartilaginous or (partially) calcified. Osteophytes visible on micro-CT are (partially) calcified; otherwise, they would not be visible. Joints were evaluated for the presence of exudate or infiltrate in the synovium, presence of hyperplasia of the synovium or fibrosis of synovium and joint capsule.

### Statistical analysis

Results are expressed as means ± SEM. Data from multiple groups were compared using one-way analysis of variance (ANOVA) followed by paired or unpaired *t*-test as appropriate. A two-way ANOVA was performed to evaluate whether there was interaction between the systemic treatment (Sham, OVX, OVX+E or OVX+BP) and IA injection. The presence or absence of osteophytes was compared using a χ^2 ^test. *P *< 0.05 was considered significant.

## Results

### Effect of oestrogen depletion and oestrogen replacement

OVX mice weighed more than Sham mice at the end of the experiment (Sham: 21.9 ± 0.6 g, OVX: 25.1 ± 0.3 g; *P *< 0.05), but no difference was found between OVX and OVX+E mice (OVX+E, 25.0 ± 1.0 g). The strong reduction in uterine weight in the untreated OVX group proved successful ovariectomy (Sham: 91.4 ± 8.4 mg, OVX: 21.8 ± 1.5 mg; *P *< 0.05). Uterus weight of OVX+E mice (OVX+E: 189.6 ± 33.5 mg) was increased compared to OVX and Sham mice.

**Figure 3:**
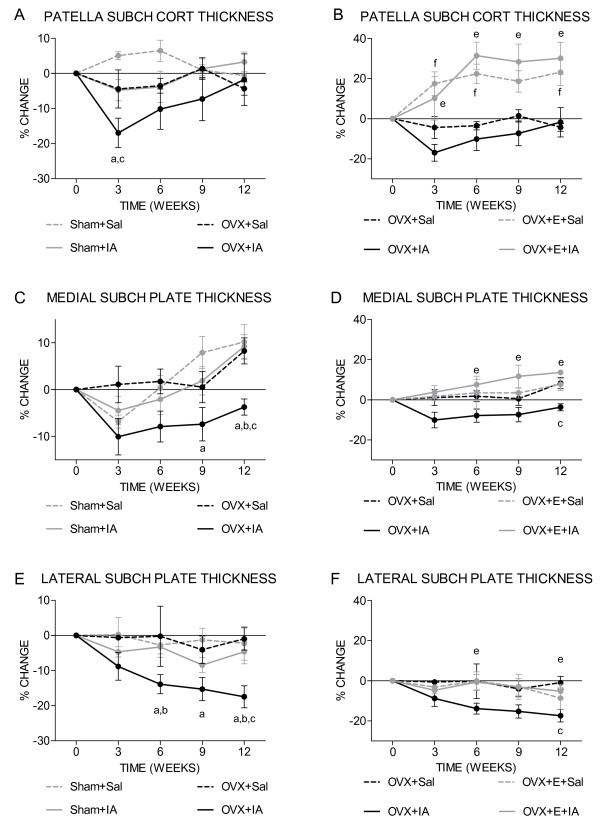
**Time course of subchondral cortical thickness**. Mice were scanned every 3 weeks in an *in vivo *micro-CT scanner. **(a **and **b) **Patella. **(b **and **c) **Medial tibia. **(d **and **e) **Lateral tibia. **(a**, **c **and **e) **Sham and OVX groups. ^a^*P *< 0.05 for OVX+IA versus Sham+Sal; ^b^*P *< 0.05 for OVX+IA versus Sham+IA, both according to unpaired *t*-test. ^c^*P *< 0.05 for OVX+IA versus OVX+Sal according to paired *t*-test. **(b, d **and **f) **OVX and OVX+E groups. ^e^*P *< 0.05 for OVX+IA versus OVX+E+IA; ^f^*P *< 0.05 for OVX+Sal versus OVX+E+Sal, both according to unpaired *t*-test. Note that in **(a)**, Sham+IA and OVX+Sal curves are overlapping up to week 9.

Bone changes were followed over time by 3-weekly micro-CT scans. In the patella, subchondral cortical thickness in OVX+IA knees was decreased at week 3, resulting from an additive effect of OVX and IA (Figure 3a). The decrease in OVX+IA was significantly different from the change in Sham+Sal and OVX+Sal mice. Subchondral cortical thickness of OVX+IA knees progressively increased from week 3 onwards, resulting in values similar to Sham+Sal, Sham+IA and OVX+Sal at week 12. Oestrogen supplementation resulted in a significantly increased subchondral cortical thickness in OVX+E+IA compared to OVX+IA knees (Figure 3b).

In the tibia, OVX+IA caused a 10% decrease in subchondral plate thickness already at week 3, but this was not significantly different from the other conditions (Figure 3c). The change in medial subchondral plate thickness in OVX+IA knees was significantly different from Sham+Sal at week 9 and from Sham+Sal, Sham+IA and OVX+Sal at week 12. This decrease in OVX+IA knees resulted from an additive effect of OVX and IA. At the lateral side, the decrease in subchondral plate thickness in OVX+IA was significantly different from Sham+Sal from week 6 onwards. At week 12, lateral subchondral plate thickness was also more decreased in OVX+IA than in Sham+IA and OVX+Sal knees (Figure 3e). Oestrogen supplementation significantly increased medial subchondral plate thickness in OVX+E+IA mice from week 6 onwards and prevented the loss in lateral subchondral plate thickness induced by OVX+IA at weeks 6 and 12 (Figures 3d and 3f).

**Figure 4:**
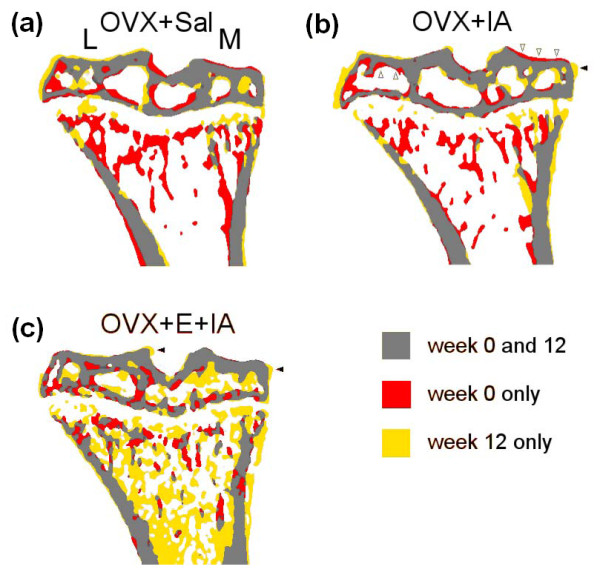
**μCT overlays visualzing bone changes in proximal tibia from start to end of experiment**. Overlaid registered longitudinal cross-section of proximal tibia scanned at start (week 0) and end of experiment (week 12) for **(a) **OVX+Sal mouse, **(b) **OVX+IA mouse, and **(c) **OVX+E+IA mouse. M indicates medial, L indicates lateral. Dark grey: present at both time points; black: only present at week 0 (i.e., resorbed in 12 weeks); light grey: only present at week 12 (i.e., newly formed in 12 weeks). Note the thinning of the subchondral plate (open arrowheads in **(b)**), loss of trabeculae in the metaphysis (both **(a) **and **(b)**), and the osteophyte formation at the medial epiphysis (closed arrowhead in **(b) **and **(c)**).

Registration software was used for the proximal tibia to compare bone images at the start (week 0) and at the end of the experiment (week 12) (Figure 4). Thinning of medial and lateral subchondral plate and osteophyte formation in the OVX+IA mouse at week 12 is clearly visible. These changes were not observed in OVX+Sal knees. In OVX+E+IA knees, medial plate thickness was increased due to endocortical apposition and osteophytes had formed.

**Figure 5:**
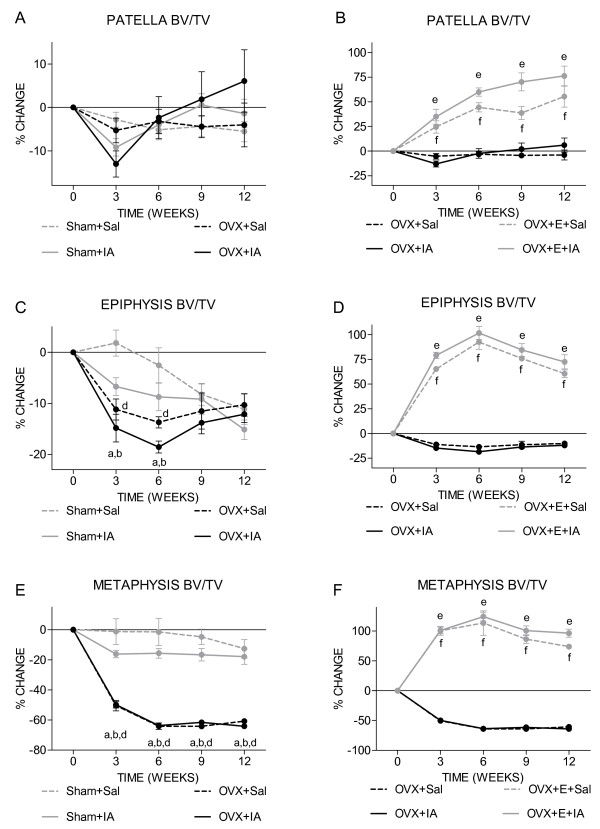
**Time course of trabecular bone volume fraction**. Mice were scanned every 3 weeks in an *in vivo *micro-CT scanner. **(a **and **b) **Patella, **(b **and **c) e**piphysis, **(d **and **e) m**etaphysis. **(a, c **and **e) **Sham and OVX groups. ^a^*P *< 0.05 for OVX+IA versus Sham+Sal; ^b^*P *< 0.05 for OVX+IA versus Sham+IA, both according to unpaired *t*-test. ^d^*P *< 0.05 for OVX+Sal versus Sham+Sal according to unpaired *t*-test. **(b, d **and **f) **OVX and OVX+E groups. ^e^*P *< 0.05 for OVX+IA versus OVX+E+IA; ^f^*P *< 0.05 for OVX+Sal versus OVX+E+Sal, both according to unpaired *t*-test. Note that OVX+Sal and OVX+IA curves are overlapping in **(e) **and **(f)**.

Patellar BV/TV was not affected in Sham+IA, OVX+Sal and OVX+IA knees (Figure 5a). Oestrogen supplementation strongly increased BV/TV in OVX+E+Sal and OVX+E+IA knees (Figure 5b). The increase in BV/TV resulted from increased trabecular bone volume as well as decreased endocortical volume as a consequence of increased subchondral cortical thickness.

Epiphyseal BV/TV decreased over time. In OVX+IA knees, the effect of IA and OVX was additive at week 3 and 6, resulting in a significantly stronger decrease than in Sham+Sal and Sham+IA. At week 12, the change in BV/TV of Sham and OVX groups was similar (-12%) (Figure 5c). In both OVX+E+Sal and OVX+E+IA, oestrogen supplementation strongly increased BV/TV compared to the OVX groups (Figure 5d). The increased BV/TV is also visible in the registered images of week 0 and 12 (Figure 4).

In the metaphysis, OVX caused a very strong decrease (-64%) in BV/TV (Figure 5e) in both OVX+Sal and OVX+IA. As in the epiphysis, oestrogen supplementation strongly increased BV/TV compared to the OVX groups (Figure 5f). These changes in metaphyseal BV/TV are also clearly visible in the registered images of weeks 0 and 12 (Figure 4). In none of the experimental conditions was metaphyseal cortical thickness affected by IA injection (data not shown).

**Figure 6:**
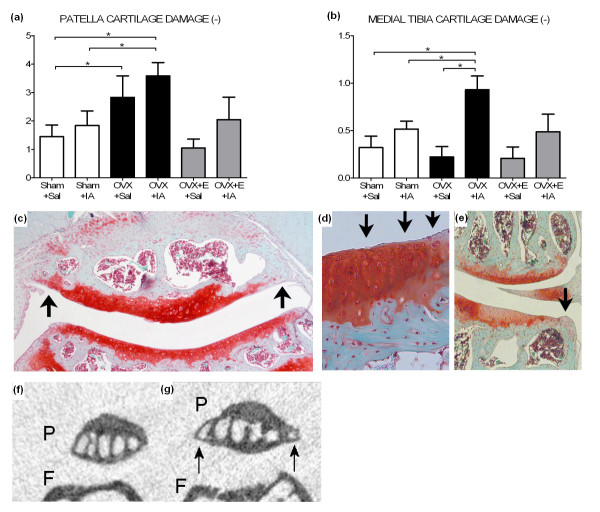
**Cartilage damage and osteophytosis at 12 weeks postsurgery for Sham, OVX, and OVX+E groups**. **(a **and **b) **Cartilage damage scored on histology using Pritzker score. **(a) **Patella cartilage damage. **(b) **Medial tibia cartilage damage. *P < 0.05 according to unpaired or paired *t*-test. **(c) **Example of patella cartilage damage and osteophytosis (arrows) on histology in an OVX+IA mouse. **(d) **Example of cartilage damage in medial tibia plateau. Arrows indicate surface fibrillation. **(e) **Example of medial knee compartment with osteophyte formation (open arrowheads). Also note glycosaminoglycan (GAG) depletion (between arrows). **(f) **Patella without osteophytes on micro-CT image. P = patella, F = femur. **(g) **Patella with osteophytes (arrows) on micro-CT image. P = patella, F = femur.

Cartilage damage was assessed on the basis of histology (Figure 6) after 12 weeks at the end of the experiment. Overall, 6 μl of 0.5% IA appeared to be a mild trigger of cartilage damage showing a clear loss of safranin O staining (data not shown). In the patella, both OVX groups had more severe cartilage damage than the Sham groups (Figure 6a). Oestrogen supplementation tended to limit the OVX-induced cartilage damage, albeit not significantly. In the tibia, cartilage damage at the medial tibia plateau of OVX+IA knees was significantly higher than that in the Sham+Sal, Sham+IA and OVX+Sal knees (Figure 6b), which was also reflected by significant interaction between OVX and IA injection. Oestrogen supplementation tended to limit medial cartilage damage caused by IA, albeit not significantly. At the lateral tibia plateau, none of the treatments had a significant effect (data not shown).

The presence of osteophytes at 12 weeks was evaluated both on histology (see examples in Figures 6c and 6e) and in micro-CT scans (see examples in Figures 6f and 6g), with the latter showing only calcified osteophytes. At the patella, more osteophytes were present after IA injection than after Sal injection. In Sham+IA and OVX+IA patellae, almost all (Sham+IA) or all (OVX+IA) osteophytes visible on histology were also visible on micro-CT, while in OVX+E+IA patellae only half of the osteophytes were also visible on micro-CT (Table 1).

**Table 1:** Presence of osteophytes on histology and micro-CT

	Patella		Medial Tibia	
		
	Histology	Micro-CT	Histology	Micro-CT
Sham+Sal	1/8 *a*	0/8 *b*	2/8 *g*	0/8 *h*
Sham+IA	6/8 *a*	5/8 *b*	8/8 *g*	5/8 *h*
OVX+Sal	2/7	0/7 *c*	6/7	0/7 *i*
OVX+IA	7/7	7/7 *c,f*	7/7	7/7 *i,j*
OVX+E+Sal	0/4 *d*	0/4	1/4	0/4
OVX+E+IA	4/4 *d*	2/4	4/4	0/4 *j*
OVX+BP+Sal	0/8 *e*	0/8	7/8	0/8 *k*
OVX+BP+IA	5/8 *e*	1/8 *f*	8/8	6/8 *k*

Osteophyte presence was scored on histology and in micro-CT data sets of the patellae and tibiae and are presented as the number of patellae/tibiae in which an osteophyte was visible on histology or micro-CT. Identical italic character indicates significant difference between these conditions according to Fisher's exact test. CT = computed tomography; Sham = sham ovariectomy; Sal = saline injection; IA = iodoacetate injection; OVX = ovariectomy; E = oestrogen supplementation; BP = bisphosphonate treatment.

At the medial tibia plateau, more osteophytes were visible on histology in Sham+IA than in Sham+Sal tibiae. On micro-CT scans, more osteophytes were visible after IA injection than after Sal injection in both Sham and OVX mice, but not in OVX+E mice. There were significantly more osteophytes visible on micro-CT in OVX+IA tibiae than in OVX+E +IA tibiae. No significant effects on lateral osteophyte presence were observed (data not shown).

No signs of exudate or infiltrate were visible after 12 weeks. OVX did not induce changes in the synovium. All joints injected with IA showed signs of mild hyperplasia, and this was not different in OVX and sham animals. None of the joints had excessive fibrosis.

### Effect of bisphosphonate treatment

To study the effect of OVX on cartilage independent of bone changes, we administered bisphosphonate (BP). Body and uterus weight of OVX+BP mice were not different from OVX mice (body weight: OVX: 25.1 ± 0.3 g, OVX+BP: 23.9 ± 1.0 g; uterus weight: OVX: 21.8 ± 1.5 mg, OVX+BP 22.8 ± 2.1 mg).

**Figure 7:**
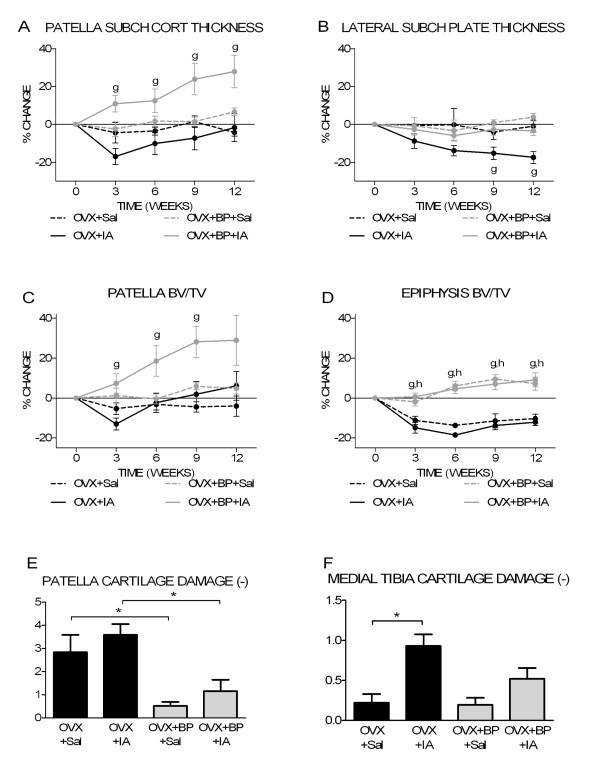
**Bone and cartilage parameters for OVX and OVX+BP groups**. **(a) **Time course of patella subchondral cortical thickness. **(b) **Time course of lateral subchondral plate thickness. **(c) **Time course of patella trabecular bone volume fraction. **(d) **Time course of epiphysis trabecular bone volume fraction. **(e) **Patella cartilage damage at 12 weeks postsurgery. **(f) **Medial tibia cartilage damage at 12 weeks postsurgery. ^g^*P *< 0.05 for OVX+IA versus OVX+BP+IA; ^h^*P *< 0.05 for OVX+Sal versus OVX+BP+Sal, both according to unpaired *t*-test. *P < 0.05 according unpaired *t*-test.

BP significantly increased patellar subchondral cortical bone thickness in OVX+IA but not in OVX+Sal knees (Figure 7a). There was significant interaction between OVX+BP and IA at weeks 3 and 9. BP compensated tibial subchondral plate thinning in OVX+IA knees at the lateral side (Figure 7b), but not at the medial side (data not shown).

BP significantly increased patellar BV/TV in OVX+IA knees (Figure 7c). This is reflected by significant interaction between OVX+BP and IA at weeks 3, 6 and 9. Similar to observations for subchondral plate thickness, BP did not affect BV/TV in OVX+Sal patellae (Figure 7c). BP increased epiphyseal and metaphyseal BV/TV in both OVX+IA and OVX+Sal tibiae at all time points (Figure 7d and data not shown).

BP significantly decreased patellar cartilage damage in both OVX+Sal and OVX+IA knees (Figure 7e). At both medial and lateral tibia plateau, BP tended to diminish cartilage damage caused by IA, albeit not significantly (Figure 7f).

In five of eight OVX+BP+IA patellae, osteophytes were detected on histology. Of these five patellae, only one had a visible osteophyte on micro-CT scans, which is significantly less than in OVX+IA patellae, where all patellae showed osteophytes on micro-CT scans (Table 1). BP did not affect osteophyte formation at the tibia plateaus.

## Discussion

The current study demonstrates that loss of oestrogen increases cartilage damage in the patella and increases susceptibility for cartilage damage in the tibia and subchondral bone loss at both sites, while oestrogen supplementation appears to dampen these effects. This observation corroborates clinical and epidemiological observations of increased OA incidence after menopause [5,8,9]. It also illustrates the interplay between hormonal changes and external triggers in the aetiology of osteoarthritis.

This study was designed to investigate the impact of oestrogen loss on articular cartilage and subchondral bone and the role of oestrogen in susceptibility of the patella and tibia for osteoarthritis, by combining a mild osteoarthritis trigger with oestrogen depletion. IA has been widely used in animal models of OA [31-34]. In both patella and tibia, injection of 6 μl of 0.5% IA did not cause a significant increase in cartilage damage. However, OVX together with IA injection led to the severest cartilage damage in the patella, indicating an increased susceptibility. At the patella, OVX alone also increased cartilage damage and supplementation with estradiol after OVX abolished this effect. Our findings are in line with previous studies that reported more cartilage damage in the tibiofemoral compartments of the knee after OVX alone [11,13,44] or after OVX in combination with OA induction [13,45,46].

In contrast to our patella data and data from the literature, in our study, tibial cartilage damage was not increased after OVX alone. However, when OVX was combined with IA injection, tibial cartilage damage was more severe than after either OVX or IA injection alone. This is reflected in the significant interaction between OVX and IA injection and further supports the hypothesis of increased susceptibility for osteoarthritis after oestrogen loss. Our data are supported by previous studies using more severe methods of OA induction in combination with OVX [13,45]. However, the effect of oestrogen supplementation was not investigated in these studies [13,45].

Subchondral cortical thickness in both the patella and tibia plateaus only decreased in the OVX situation when the osteoarthritis trigger had been applied. The effects of OVX and IA treatment were additive, resulting in a significantly decreased subchondral cortical thickness. This provides further support for a role of estradiol in osteoarthritis and adds to the concept of the involvement of bone, specifically the subchondral bone plate, in osteoarthritis. It remains unknown whether these changes are directly caused by the osteoarthritis process triggered by IA or are caused by unloading of the joint (due to pain); it has been suggested that oestrogen deficiency may exacerbate pain perception [47]. However, cortical bone in the metaphysis was not affected, suggesting that changes in subchondral cortical bone of the tibia are not caused by unloading, but are an intrinsic part of the osteoarthritic pathological process.

The decrease in subchondral plate thickness is in line with findings from previous OA animal studies evaluating the early disease stage [26,27,48-50]. In some of these studies, this was followed by plate thickening [26,49]. This also explains the discrepancy with sclerosis described in human studies [51-53], which often concern patients with late osteoarthritis, whereas our present study reflects an early phase of osteoarthritis. Comparison of patella and medial and lateral tibia demonstrated an as yet unexplained site specificity. At the patella and tibial medial side after 3 weeks of OVX-IA, the subchondral plate showed a progressive restoration of thickness, while at the lateral tibia side a progressive thinning was observed (compare OVX+IA in Figures 3a, c and 3e).

Loss of estradiol did not cause changes in patellar trabecular bone, and compared to the metaphysis, caused only mild changes in epiphyseal trabecular bone. However, at all three sites, supplementation with estradiol strongly increased bone volume fraction, suggesting that these sites are sensitive but that patellar and epiphyseal bone are less dependent on circulating estradiol than metaphyseal bone. This may be due to differences in oestrogen receptor levels or in local estradiol production. These as yet unknown differences between epiphyseal and patellar bone on one side and metaphyseal bone on the other side could be important for understanding the functional link between estradiol loss, bone turnover and development of osteoarthritis.

To further investigate the relationship of bone changes with cartilage damage, we studied bone and cartilage changes in OVX mice in the absence or presence of BP. In this way, we attempted to assess the impact of OVX on the IA effect on cartilage while the changes in bone would be blocked. In the tibia, treatment with BP prevented subchondral plate thinning after IA injection and increased bone volume fraction in both saline-injected and IA-injected epiphyses. Both medial and lateral cartilage damage appeared to be less when OVX-IA mice were treated with BP. In the patella, the effect of BP treatment was different. In OVX+BP+Sal patellae, no bone changes occurred, while in OVX+BP+IA patellae, subchondral cortical thickness and bone volume fraction were strongly increased (Figures 7a and 7c). Because this occurred only in IA-injected knees, IA may have activated bone turnover (increased activity of both osteoblasts and osteoclasts), of which BP only reduced osteoclastic resorption, resulting in a net increase. In contrast, estradiol is also directly bone anabolic, explaining the increase in bone volume in both OVX+E+Sal and OVX+E+IA groups (compare with Figures 5b, d and 5f). Interestingly, cartilage damage was less in both BP groups (OVX+BP+Sal and OVX+BP+IA), which is in line with reports suggesting that BPs may have an OA protective effect [54-56]. Since the decrease in patellar cartilage damage occurred independently of the observed bone changes in both BP groups, BP may have a direct effect on cartilage. This precludes drawing definitive conclusions on the relation between bone changes and cartilage changes.

It has been suggested in the literature that patella OA is distinct from tibiofemoral OA [4]. When comparing patellar changes to tibial changes, we notice several differences between these compartments. First, cartilage damage is more severe in the patella than in the tibia, which is in line with previous reports [57,58]. Second, oestrogen depletion increases cartilage damage in the patella, but increases the susceptibility for cartilage damage in the tibia. Third, subchondral cortical bone became thinner in the first 3 weeks in both areas (patella and tibia) after OVX+IA, but in the tibia the thickness continued to be decreased, while in the patella the thickness subsequently increased to values similar to Sham patellae. This may reflect different dynamics or a different mechanism, which is supported by differences in biochemical and mechanical properties of articular cartilage of patella and tibia [59-61] and by differences in biomechanics between the patellofemoral and tibiofemoral joint [4].

## Conclusions

These data demonstrate the significance of oestrogen for articular cartilage and subchondral bone and the maintenance of healthy joints. Depending on the site in the joint, oestrogen depletion may directly increase cartilage damage and subchondral bone loss or increase susceptibility for an additional trigger. The current data support an etiological role for altered oestrogen signalling in osteoarthritis and thereby substantiates the link between estradiol and the development of osteoarthritis. The current data strongly add to the concept of the involvement of bone, specifically the subchondral bone plate, in osteoarthritis.

## Abbreviations

BP: bisphosphonate; BV/TV: bone volume fraction; Conn D: connectivity density; CT: computed tomography; E: oestrogen; ER: oestrogen receptor; GAG: glycosaminoglycan; IA: iodoacetate; OA: osteoarthritis; OVX: ovariectomy; PFJ: patellofemoral joint; PlTh: subchondral plate thickness; ROI: region of interest; Sal: saline; SMI: structure model index; TbTh: trabecular thickness; TFJ: tibiofemoral joint.

## Competing interests

The authors declare that they have no competing interests.

## Authors' contributions

Yvonne Sniekers carried out the experiments and analyses and drafted the manuscript. All other authors were involved in the design of the study, interpretation of the data and revision of the manuscript. All authors read and approved the final manuscript.
